# Determinants of bone health in adults Polish women: The influence of physical activity, nutrition, sun exposure and biological factors

**DOI:** 10.1371/journal.pone.0238127

**Published:** 2020-09-22

**Authors:** Anna Kopiczko

**Affiliations:** Department of Biomedical Sciences, Józef Piłsudski University of Physical Education in Warsaw, Warsaw, Poland; Addis Ababa University School of Public Health, ETHIOPIA

## Abstract

**Purpose:**

The aim of this study was to assess the determinants of bone health in the group of women over 40 years old. Lifestyle factors such as past and present physical activity, past and present sun exposure, current dietary intake of calcium and vitamin D, nutritional status as measured by BMI, family history of osteoporosis and current hormonal status were analysed.

**Methods:**

The study involved 500 women over 40 years old. All examined women was the same ethnicity- European origin. Methods used: densitometry method (DXA), bioelectrical impedance analysis, International Physical Activity Questionnaire, nutrition questionnaire, past and present sun exposure questionnaire. Past and present physical activity, past and present sun exposure and sufficient level of calcium in the diet proved to be the most important factors determining mineralization of bone tissue of women. In order to indicate an independent association of the correct bone tissue mineralization with individual factors, multivariate analysis was used—logistic regression.

**Results:**

The norm BMD in the distal part of the forearm was strongly influenced: recommended dietary calcium intake (OR = 5.95; p = 0.003), moderately (OR = 1.88; p = 0.053) and high (OR = 14.0; p<0.001) past physical activity, sufficient (OR = 4.97; p<0.001) and high (OR = 18.9; p = 0.004) level of present physical activity, sufficient past (OR = 5.15; p<0.001) and sufficient present sun exposure (OR = 10.0; p<0.001).

The chance for the BMD prox norm was also increased several times: high past physical activity (OR = 68.4; p<0.0001) and sufficient past sun exposure (OR = 10.6; p<0.001), moderate past activity (OR = 4.20; p<0.001), sufficient (OR = 6.13; p<0.001) and high (OR = 10.0; p<0.001) present physical activity, sufficient present sun exposure (OR = 9.09; p<0.0001), recommended intake of calcium (OR = 9.57; p<0.001) and vitamin D (OR = 2.68; p = 0.052). Whereas e significantly lower likelihood for the BMD prox norm was found in women with the oldest hormonal status (postmenopausal period) (OR = 0.18; p<0.001), with osteoporosis in the family (OR = 0.37; p<0.001) and living in an agglomeration (OR = 0.68; p = 0.03).

**Conclusion:**

Interventions to increase physical activity, especially outdoors, may help reduce risk of osteoporosis, fractures and subsequent healthcare costs.

## Introduction

Low bone mineral density is the strongest risk factor for osteoporosis and related fractures. Osteoporosis has been diagnosed mostly in postmenopausal women, but the onset of the disease occurs at a much earlier age. Low peak bone mass and low bone mineral density in relation to age and gender are increasingly common in the population of young women and men [1, 2].

In addition to genetic determinants [3], other bone health determinants whose effects and interactions still need to be explored include lifestyles and general health status. Smoking and alcohol consumption, sedentary lifestyles and long-term use of some drugs [4] and coexisting diseases [5] may significantly determine the mineral status and skeletal strength. Furthermore, the physiological condition, age, body weight, body height, fat-free mass and hormonal status in women are also critical to bone parameters [6–8],

Several cross-sectional studies have shown that bone mineral density is strongly affected by genetics [9]. The results of epidemiological observations document the family burden of osteoporosis. A stronger tendency to intensive osteoporosis was demonstrated in women coming from families with a history of this disease [10]. The probability of osteoporosis in a woman whose mother had osteoporosis was higher the more similar the type of body structure and body composition was demonstrated between mothers and daughters [11]. Heredity, i.e. the additive effect of genes and their polymorphisms is estimated at 50–80% of the variability of bone mass and bone structure [12].

Loss of bone mineral content (BMC) in women occurs mainly in the postmenopausal period. This is due to disorders of the equilibrium between bone resorption processes (action of osteoclasts) and bone-forming processes (action of osteoblasts).

Epidemiological studies have shown that the frequency of bone fractures due to osteoporosis after menopause is higher than the number of heart attacks, strokes and even breast cancer. These diseases can result in significant direct (medical treatment) and indirect (workplace) costs. Osteoporosis takes a huge personal and economic toll. In Europe, the disability due to osteoporosis is greater than that caused by cancers and is comparable or greater than that lost to a variety of chronic noncommunicable diseases, such as rheumatoid arthritis, asthma and high blood pressure related heart disease [13].

Femoral neck fractures are becoming more frequent as the world's population ages. It is estimated that the frequency of femoral neck fractures increases by 1 to 3% annually in most regions of the world [14]. It was estimated that the number of patients worldwide with osteoporotic hip fractures is more than 200 million. It was reported that in both Europe and the United States, 30% women are osteoporotic, and it was estimated that 40% post-menopausal women will experience an osteoporotic fracture in the rest of their lives [15,16].

From the standpoint of public health, osteoporotic fractures generate high costs of treatment and can result in early disability and even lead to death. Since there are no early symptoms of osteoporosis, the assessment of the risk of low bone mass and bone mineral density at the age before older adulthood is crucial.

Convincing evidence indicates that regular physical activity, especially weight training and calisthenics, positively affects the bone metabolism of women irrespective of age [17]. The beneficial effect of regular exercises on the skeleton results from the necessary axial load, which favours bone formation and its proper remodelling [18]. An active lifestyle at the age of the development of peak bone mass continued in later stages of ontogenesis is an optimal solution that ensures bone health in older adulthood.

In addition to physical activity, studies indicate a number of other factors influencing bone parameters. The most important factors include the history of fractures in the family, low body mass index and poor body musculature, deficiencies of calcium, protein and vitamin D in the diet, lack of active forms of vitamin D from solar photoconversion. In total, these factors account for over 40% of BMD variability [19, 20].

An important risk factor of osteoporosis is insufficient sun exposure, which, in addition to nutrition, is an important source of vitamin D [21, 22]. In order for the cutaneous synthesis of vitamin D to be effective, it is necessary to uncover a suitable body surface area and ensure the skilful and healthy use of sunbathing. Furthermore, in the northern latitudes, negligible UVB radiation in winter sunlight is observed, which means that vitamin D must be produced and accumulated in the skin during the summer months [23]. There are few data on sun exposure in terms of bone health.

Then a healthy diet is of great importance for the proper development of the skeletal system and the prevention and treatment of osteoporosis in all populations. It represents an important modifiable factor affecting bone health [24]. Nutrients are structural bone components. A balanced diet and skilful use of dietary supplements provide essential nutrients to bone tissue, such as calcium, protein, vitamin D and K [25].

The aim of this study was to assess the determinants of bone health in the group of women over 40 years old. Lifestyle factors such as past and present physical activity, past and present sun exposure, current dietary intake of calcium and vitamin D, nutritional status as measured by BMI, family history of osteoporosis and current hormonal status were analysed.

## Materials and methods

### Study population

The study involved 500 women (250 women from a small town Augustow and 250 women from agglomeration Warsaw. All women studied were of the same ethnic origin (European). The sample size was calculated using the formula [26], taking into account individual variability in BMD and standard error, taken from published studies on biological condition of Polish women of similar age. The drawing was carried out from the collection of data obtained from the population register of City Councils (random-systemic selection, recruitment every fifth woman on the register list in age category) to get 500 women. The first environment is a small town with 30.000 inhabitants. The city has a touristic character. The second place is a large agglomeration, the capital of the country with 1 million 700 thousand inhabitants. The research was of a screening character. The sample was random so it can be considered as representative of the population of women in a small town. Research on the population of women from a large agglomeration will continue. The study was conducted from May to September 2012. The study of women from Warsaw was conducted in the research laboratory of the Department of Anthropology and Health Promotion in Józef Piłsudski University of Physical Education in Warsaw. The examination of women from Augustow required transporting all the research equipment and preparing the examination laboratory in the city medical clinic. It is planned to repeat the survey in 2020 to assess changes in BMD and BMC. All measurements and interviews were carried out in adapted testing laboratories. All women included in the study were informed about the aims and schedule of the study. The study involved women who were invited, have given their written consent to participate and did not have the diseases described in the exclusion criteria. The exclusion criteria included hormone replacement therapy, kidney disease, thyroid and parathyroid diseases, cancers, rheumatoid arthritis, and long-term steroid treatment [6].

The project received a positive opinion on the compliance with the rules of ethics of the of the Senate Ethics Committee for Scientific Research of the Józef Piłsudski University of Physical Education in Warsaw protocol number SKE 01-10/2011. The study was carried out in accordance with the principles and provisions of the Declaration of Helsinki.

### Somatic measurements and body composition

The anthropometric measurement protocol described by Hall et al. [27] was used. Body mass was measured using the First Australia scales with an accuracy of 0.1 kg. Body height (basis-vertex) was measured by a qualified anthropologist. During the measurements, the study participants were wearing light underwear and were barefoot. Body height was measured two times with an anthropometer (GPM, Siber Hegner, Zurich, Switzerland) with an accuracy of 1mm. The measurement error it was 0.22 cm. The forearm length was measured using callipers at the radiale-stylion points (r-sty). Body mass index (BMI) was calculated. Fat mass (FM) and fat-free mass (FFM) were analysed using the JAWON Medical x-scan device based on bioelectrical impedance analysis. All measurements were performed according to the applicable methodology and with the same measurement instruments.

### Assessment of hormonal status

Hormonal status was rated based on a self-assessment questionnaire. We used hormonal status classification recommended by the World Health Organization [28]. Premenopause is the period before menopause characterized by rhythmic menstrual cycles). Perimenopause is the period immediately preceding menopause, in which menstrual cycles are longer and irregular). Postmenopause is natural menopause. Information on the occurrence of osteoporosis in the family was collected in a direct interview [6].

### Bone tissue measurement method and family history of osteoporosis

Bone mineral density (BMD in g/cm^2^), bone mineral content (BMC in g) and T-scores of two points (distal and proximal) of the non-dominant forearm were measured using dual-energy X-ray absorptiometry method with the Norland instrument. DXA apparatus was used to measure the peripheral skeleton, regional measurement. The bone examination was performed once. The effective dose (μSv) for this densitometer is 0.05. The densitometry data were used to calculate the T-score. The World Health Organization definition of osteoporosis is based on the T-score [29]. The T-score is a comparison of a patient’s BMD to that of a healthy thirty-year-old of the same sex and ethnicity (expressed in standard deviations). Bone parameters were measured according to the adopted densitometry methodology and the recommendations of the International Society for Clinical Densitometry [30]. According to the recommendations of the manufacturer of DXA Norland, the scanner was calibrated daily. We used a standard calibration block. The examination was performed by a team with the certificates needed to perform peripheral and axial densitometry and experienced in densitometry. Information on the occurrence of osteoporosis in the family was collected in a direct interview. The questions also concerned the occurrence of osteoporotic fractures in the family [6].

### Method of measuring physical activity

For evaluation of physical activity (PA), we used questionnaires for two methods: retrospective (for the past level of PA it was related to adolescence, which is the period of peak bone mass) and prospective (for current habitual PA). Information about past PA was collected during a direct interview. We used the following classification: inactive women (inactivity during and after PE classes at school), moderately active women (participation in PE classes but inactivity in leisure time) and women with a high level of physical activity (regular sports training) [6]. For evaluation of current PA, we used the International Physical Activity Questionnaire -Short Form (IPAQ). Activities undertaken throughout the week were taken into account. The intensity of activities was determined using the metabolic equivalent of task (MET). The classification of physical activity level was based on the recommendations of the American College of Sports Medicine and WHO [6, 31–33].

### Method of measuring dietary intake of calcium and vitamin D

A questionnaire was used to evaluate dietary intake of calcium and vitamin D in the daily food ration (mg or μg/person/day) according to the guidelines for the group intake assessment frequencies and quantities of consumption in the last month preceding the survey. Nutrition from both weekdays and weekends was analysed. The method used was a dietary interview (standardized questionnaire to evaluate food consumed in terms of products critical to calcium and vitamin D intake) developed in the Department of Epidemiology Institute of Food and Nutrition in Warsaw. A face-to-face nutritional interview was conducted by a qualified nutritionist. Nutrient intake was calculated using the computer software Diet 5.0 (extended version). The amount of calcium and vitamin D intake per day was related to the dietary standards for the population. In accordance with expert recommendations and Diet 5.0 computer software, the results were related to Adequate Intake- AI standard. On this basis, women's diets were classified as Deficiency or Recommended intake levels.

### Method of assessment sun exposure

The information about past and present sun exposure (SA) was obtained using a questionnaire prepared in cooperation with experts in vitamin D in Poland. The sunshine questionnaire was conducted during the September summer visit when the UV solar radiation is highest.

The examination concerned the period from April to September, which is the period when, due to the specific geographical zone, skin synthesis is observed [22].

The questionnaire consisted of 10 questions regarding two categories. The questions were inspired by the international OPTIFORD study. The frequency category concerns questions about the number of days of sun use in the summer months. Time and criteria for the sun exposure are questions about the time spent in the sun, which hours, how many minutes, the assessment of body surface exposure to UV radiation, the usage of sunscreen. Answers were given in five categories from several times a day, every day, several days a week, and the answer: no exposure. The categories of sufficient and insufficient exposure were developed using the method of summing points from the data obtained from the questionnaire: the points of the two categories of the frequency and sun exposure time and criteria were summed up. Sufficient sun exposure was adopted in accordance with the guidelines and recommendations for the prevention of vitamin D deficiency developed by a group of experts [22, 34]. The questionnaire was pre-tested in pilot study [22].

### Statistical methods

All statistical analyses were performed using the STATISTICA software program (v. 11, StatSoft, USA). The Shapiro‐Wilk test was applied to examine the distribution of the results. The conducted analysis confirmed the assumption of normality of the distribution. Univariate and multivariate models of logistic regressions were used to assess the probability of the occurrence of normal BMD in the distal and proximal part of the forearm bone according to the analyzed variables. Multivariate analyses were conducted using the backward stepwise procedure. For the independent variables, odds ratios (OR) with 95% confidence intervals (CI) were calculated, and the Nagelkerke R2 was estimated as a measure of goodness-of-fit. The statistical significance was set at *α <0.01; **α <0.001 for all analyses.

## Results

### Characteristics of the study subjects

The general characteristics of 500 women divided by the place of residence are shown in Table 1. Women from a small town had statistically significantly higher fat-free mass (FFM), almost two times higher MET/week, bone mineral content (BMC) in the proximal part of forearm (p<0.05), and higher calcium and vitamin D intake in the diet compared to women from a large agglomeration (p<0.001). Furthermore, they had significantly smaller values of BMD and T-score in the distal part (p<0.05) (Table 1).

**Table 1:** General characteristics of the analysed variables in women.

Variables	Small town (n = 250)		Agglomeration (n = 250)		Student's t-test	
	mean	SD	mean	SD	T	*p*
Age [years]	51.9	7.8	52.0	7.7	-0.142	0.887
Weight [kg]	72.8	12.3	71.2	13.9	1.373	0.171
Height [cm]	162.4	5.2	163.1	6.1	-1.360	0.174
BMI [kg/m^2^]	27.6	4.6	26.8	5.1	1.980	0.057
FM [kg]	25.9	8.8	25.8	10.0	0.097	0.923
FFM [kg]	47.0	4.5	45.5	4.8	3.577	0.001
BMD dis [g/cm^2^]	0.348	0.07	0.361	0.08	-2.125	0.034
BMD prox [g/cm^2^]	0.737	0.09	0.728	0.09	1.184	0.237
BMC dis [g]	1.380	0.25	1.370	0.26	0.427	0,669
BMC prox [g]	1.921	0.25	1.876	0.26	1.987	0.047
T-score dis	-0.018	1.07	0.229	1.495	-2.127	0.034
T-score prox	-1.282	1.40	-1.408	1.46	0.991	0.322
MET [min/week]	1374.6	1505.4	645.6	871.1	6.590	0.001
Calcium [mg/day]	456.8	436.3	371.7	515.0	1.994	0.047
Vitamin D [μg/day]	3.32	3.61	2.17	2.28	4.265	0.001

BMI–Body Mass Index, FM- Fat Mass, FFM- Fat Free Mass, BMD–bone mineral density, BMC–bone mass, MET- Metabolic Equivalent Task

### Prevalence of low BMD and the findings of the logistic regression analyses

Low BMD in the distal section of the forearm occurred with similar frequency in women from small town and agglomeration. The results obtained from the logistic regression model indicate a significant strong impact on the BMD standard in the distal part of the forearm of such variables as: recommended dietary calcium intake (OR = 5.95; p = 0.003), moderately (OR = 1.88; p = 0.053) and high (OR = 14.0; p<0.001) past physical activity, sufficient (OR = 4.97; p<0.001) and high (OR = 18.9; p = 0.004) level of present physical activity, sufficient past (OR = 5.15; p<0.001) and sufficient present sun exposure (OR = 10.0; p<0.001). Older hormonal age (postmenopause) significantly reduced the chance of BMD dis norm (OR = 0.06;p<0.001), (Table 2).

**Table 2:** Results of logistic regression analysis—Odds Ratios (OR) calculated for a normal BMD in the distal part of the forearm bone.

Variable		Low BMD n = 91	Normal BMD n = 409	Univariate regression models		Multivariate regression model^a^
				OR crude (95%CI)	R^2^ Nagelkerke	OR adjusted (95%CI)
**Place to live**	Small town	46.2	50.9	1	0.002	Not included in the model
	Agglomeration	53.8	49.1	0.83 (0.53–1.31)		
**BMI**	Norm	36.7	37.3	1	0.011	Not included in the model
	Overweight	44.4	35.8	0.79 (0.47–1.32)		
	Obesity	18.9	27.0	1.40 (0.74–2.65)		
**Biological age**	Perimenopause	3.3	31.1	1	0.211	1
	Premenopause	4.4	20.3	0.49 (0.11–2.24)		0.42 (0.09–2.03)
	Postmenopause	92.3	48.7	0.06** (0.02–0.18)		0.10** (0.03–0.32)
**Calcium (mg/day)**	Deficiency	96.7	83.1	1	0.047	Not included in the model
	Recommended intake	3.3	16.9	5.95* (1.83–19.4)		
**Vitamin D (μg/day)**	Deficiency	96.7	96.3	1	<0.001	Not included in the model
	Recommended intake	3.3	3.7	1.12 (0.32–3.94)		
**Osteoporosis in the family**	No	82.4	85.0	1	0.001	Not included in the model
	Yes	17.6	15.0	0.82 (0.45–1.51)		
**Past PA**	Inactive	18.7	8.3	1	0.102	Not included in the model
	Moderately active	76.9	64.3	1.88 (0.99–3.56)		
	High	4.4	27.4	14.0** (4.41–44.4)		
**Present habitual PA**	Insufficient	83.5	46.2	1	0.148	1
	Sufficient	15.4	42.3	4.97** (2.71–9.11)		2.38* (1.22–4.64)
	High	1.1	11.5	18.9* (2.56–139)		7.95 (1.03–61.5)
**Past SA**	Insufficient	35.2	9.5	1	0.104	1
	Sufficient	64.8	90.5	5.15** (2.99–8.85)		2.51* (1.37–4.58)
**Present SA**	Insufficient	91.2	50.9	1	0.180	1
	Sufficient	8.8	49.1	10.0** (4.73–21.2)		4.39** (1.97–9.79)

BMD–Bone mineral density; PA- physical activity; SA- sun exposure; a—R2 Nagelkerke for the multivariate regression model = 0.359; The statistical significance

*p<0.01

**<0.001

An analogous analysis with logistic regression was performed for the proximal section. The highest and strongly significant odds ratio for BMD prox norm was determined by high past physical activity (OR = 68.4; p<0.0001) and sufficient past sun exposure (OR = 10.6; p<0.001). Moderate past activity increased the chances of good mineralization in the proximal part of the forearm by more than six times (OR = 4.20; p<0.001). The chance for the BMD prox norm was also increased several times: sufficient (OR = 6.13; p<0.001) and high (OR = 10.0; p<0.001) present physical activity, sufficient present sun exposure (OR = 9.09; p<0.0001), recommended intake of calcium (OR = 9.57; p<0.001) and vitamin D (OR = 2.68; p = 0.052). Whereas e significantly lower likelihood for the BMD prox norm was found in women with the oldest hormonal status (postmenopausal period) (OR = 0.18; p<0.001), with osteoporosis in the family (OR = 0.37; p<0.001) and living in an agglomeration (OR = 0.68; p = 0.03), (Table 3).

**Table 3:** Results of logistic regression analysis—Odds Ratios (OR) calculated for a normal BMD in the proximal part of the forearm bone.

Variable		Low BMD n = 91	Normal BMD n = 409	Univariate regression models		Multivariate regression model^a^
				OR crude (95%CI)	R^2^ Nagelkerke	OR adjusted (95%CI)
**Place to live**	Small town	45.7	55.5	1	0.013	Not included in the model
	Agglomeration	54.3	44.5	0.68 (0.47–0.96)		
**BMI**	Norm	36.1	38.5	1	0.012	1
	Overweight	41.1	32.6	0.74 (0.49–1.12)		0.84 (0.45–1.55)
	Obesity	22.9	28.9	1.18 (0.75–1.86)		2.68* (1.39–5.13)
**Biological age**	Perimenopause	16.0	39.0	1	0.211	1
	Premenopause	9.6	27.5	1.18 (0.66–2.10)		0.97 (0.44–2.15)
	Postmenopause	74.5	33.5	0.18** 0.12–0.29		0.35** (0.19–0.63)
**Calcium (mg/day)**	Deficiency	96.1	72.0	1	0.153	1
	Recommended intake	3.9	28.0	9.57** (4.89–18.7)		3.87** (1.69–8.89)
**Vitamin D (μg/day)**	Deficiency	97.9	94.5	1	0.011	Not included in the model
	Recommended intake	2.1	5.5	2.68 (0.99–7.26)		
**Osteoporosis in the family**	No	79.4	91.3	1	0.037	1
	Yes	20.6	8.7	0.37** (0.21–0.64)		0.47 (0.22–0.97)
**Past PA**	Inactive	16.7	1.8	1	0.309	1
	Moderately active	77.3	52.8	6.20** (2.18–17.6)		4.70 (1.28–17.3)
	High	6.0	45.4	68.4** (21.8–214)		28.1** (6.78–116.7)
**Present habitual PA**	Insufficient	72.3	28.0	1	0.248	1
	Sufficient	23.4	55.5	6.13** (4.05–9.28)		3.36** (1.95–5.77)
	High	4.3	16.5	10.0** (4.92–20.5)		4.77** (2.03–11.2)
**Past SA**	Insufficient	23.0	2.8	1	0.126	1
	Sufficient	77.0	97.2	10.6 ** (4.49–24.9)		3.22 (1.10–9.43)
**Present SA**	Insufficient	79.8	30.3	1	0.304	1
	Sufficient	20.2	69.7	9.09** (6.03–13.7)		2.85** (1.68–4.85)

BMD–Bone mineral density; PA- physical activity; SA- sun exposure; a—R2 Nagelkerke for the multivariate regression model = 0.359; The statistical significance

*p<0.01

**<0.001.

## Discussion

In this study, we found a significantly strong effect on the BMD norm in the distal part of the forearm of such variables as younger biological age (premenopausal hormonal status), sufficient and high present physical activity, sufficient past and present sun exposure. In the proximal part of the forearm, the highest and strongly significant odds ratio for BMD prox norm was determined by high past physical activity and high current habitual physical activity. Moderate past activity increased the chances of good mineralization in the proximal part of the forearm by more than six times whereas sufficient past and present sun exposure increased these chances by several times. The recommended calcium and vitamin D intake significantly affected the BMD prox norm.

Some studies have shown that increased BMI has a protective effect on bone density, whereas moderately overweight individuals were found to be characterized by increased BMD, indicating that BMI and weight gain may be related to BMD. More importantly, increased body weight has been shown to correlate with hormonal changes, which may positively affect bone metabolism in women [35]. The significantly lower likelihood for the BMD prox norm was found in women with the oldest hormonal status (postmenopausal period). Osteoporosis is the most common disease among metabolic bone diseases. It weakens bone mass by destroying bone microstructure. As women get older, they lose 30–50% of trabecular bone mass and 25–30% of cortical bone mass, with the largest volume of bone mass lost in the pre-menopausal and postmenopausal periods [7,19]. The rate of bone density loss varies depending on the location of the skeleton. Therefore, BMD is tested at various bone points. In this study, BMD and BMC were higher in the proximal forearm, where the bone is thicker.

This study found a significantly strong effect on the BMD norm in the distal and proximal parts of the forearm of sufficient and high levels of habitual physical activity. In the proximal forearm, the highest and strongly significant odds ratio for BMD prox norm was determined by high past physical activity. A moderate level of active participation in physical education classes increased the chances of good forearm mineralization in the proximal forearm more than six times. High physical activity in the past and practising various sports increased the chances for normal BMD more than sixty times.

Kumar et al. [36], in a two-year study of menopausal women concluded that physical activity can be regarded as one of the important predictors of BMD. An important determinant of bone health and normal BMD in both the lumbar spine and the femoral neck was primarily the physical activity of women. Cross-sectional studies of women with menopausal status confirmed this thesis. It was demonstrated that BMD in the femur and lumbar spine was significantly higher in physically active women, showing the largest metabolic equivalent of task [37].

Physical exercise is considered an effective factor in stimulating bone osteogenesis. There is evidence that exercise induces an increase in bone mass in younger subjects. In older adults, the results of the studies indicate that exercise may increase the thickness and resistance of the cortical bone at loaded skeletal sites [38]. Therefore, there is considerable interest in defining the adequate dose and characteristics of exercises to improve bone strength in osteoporosis and to develop appropriate practical guidelines.

In this study analysed past and present sun exposure. The human body is capable of endogenous synthesis of cholecalciferol 7-dehydecholesterol in skin cells using ultraviolet radiation. Therefore, the efficiency of this process depends on skin pigmentation, age, time and frequency of sun exposure, and latitude in which the person lives. Vitamin D deficiency in the human body can be caused by insufficient endogenous synthesis or insufficient intake of this vitamin in the diet, causing a higher risk of osteoporosis [39].

In our study, a sufficient level of past and present sun exposure in women significantly increased the chances of good forearm mineralization (OR>5). A study on the level of sun exposure showed that sun exposure during the summer peak in around one-quarter of teenagers is insufficient [23]. Seasonal vitamin D deficiency is common, and those affected usually had low BMD. Similarly, in a study of women, half of the respondents reported only occasional sun exposure [22].

Lack of regular sun exposure, especially in older adults, may be caused by fear of sunburn and skin cancers. Sunscreens can be used as protection against solar radiation. However, it should be noted that the commonly used sunblocks can reduce the efficiency of skin synthesis due to UVB radiation by up to 90%. Research shows that education in the safe use of sunbathing is needed.

The present study of Polish women did find the relationship between BMD and the level of vitamin D in the diet only with BMD prox. The recommended calcium intake was associated with a significant odds ratio (OR >5) for BMD norm in the distal forearm and in prox part more than nine (OR = 9.56).

One new meta-analysis suggested that a healthy dietary regimen is likely to reduce the risk of low BMD among children, adolescents, young adults and older adults regardless of gender. Nutrients, especially calcium, vitamin D, phosphorus, potassium, magnesium, and vitamin K and some food groups have shown beneficial effects on bone health and lower risk of fracture [40, 41].

In this study, only calcium and vitamin D in the diet were analysed, which represents the limitation of the study. The study demonstrated that the intake of calcium and vitamin D was higher among women from Augustów, which is a small town, compared to those from a large agglomeration.

Many studies have evaluated the food intake of women living in rural and urban areas [42]. A study carried out in Australia [43] reported a better quality of diets in urban women compared to their rural peers, whereas other studies carried out in Greece and Africa showed opposite results [44].

Differences in data collection methodologies, assessment of diet quality, urban and rural environment definitions and socio-economic differences between developed and developing countries may partly explain the differences reported. However, it is still emphasized that the rural population continues to consume more milk products with high calcium and protein content and more healthy agricultural products [45]. A recent study on the nutrition of Polish women showed that most of the diets studied did not meet the health criteria included in the pro-Healthy Diet Index (pHDI-10) [46]. Therefore, it is recommended to develop culturally acceptable and appropriate interventions.

In this study, osteoporosis in the family, especially suffered by mothers, did not lead to a low chance for normal BMD dis. However, a significantly lower chances for norm BMD in proximal part of forearm was found in women with a family history of osteoporosis.

Perhaps BMD in these studies was more significantly affected by lifestyle-related variables. It should be added that no cases of osteoporotic fractures were found among the mothers of the examined women.

The study Sobas et al. carried out in 2007–2010 on 712 pairs of mothers and daughters showed that the presence of bone fracture risk factors in mothers and daughters was significantly correlated [47].

Robitaille et al. [48] obtained information on osteoporosis in first-degree relatives and grandparents during interviews with 8073 women aged 20 years and older. These findings indicate that family history is a significant independent risk factor for osteoporosis in women aged 35 years and older. Further research is needed to assess family history as a convenient and inexpensive tool to identify women at risk of osteoporosis and to promote preventive behaviours.

The study has some limitations. The research was conducted in two regions of Poland. Screening in all regions would be of great value. This project did not determine biochemical blood indicators, assessment of nutrition, calcium, protein and vitamin D intake in diet and this would give the full status of the BMD determinants. The examination concerned only forearm bones. DXA studies of the spine and femoral neck would provide a complete picture of the condition of bone tissue.

## Conclusions

The most important factors determining the statistically significant healthy mineralization of bone tissue in women are an adequate dose of past and present physical activity, sufficient past and present sun exposure and sufficient calcium vitamin D intake in the diet.

It is recommended to develop educational programs to promote the role of physical activity throughout life (especially practised outdoors) and sufficient sun exposure, and their effects on the development of adequate mass and optimal mineralization of bones in women.

Interventions to increase physical activity, especially outdoors, may help reduce risk of osteoporosis, fractures and subsequent healthcare costs.

## Supporting information

S1 Data (XLSX)

S1 Table Results of logistic regression analysis—Odds Ratios (OR) calculated for a normal BMD in the distal part of the forearm bone. (DOCX)

S2 Table Results of logistic regression analysis—Odds Ratios (OR) calculated for a normal BMD in the proximal part of the forearm bone. (DOCX)
