# Supplementary material for: Determinants of bone health in adults Polish women: The influence of physical activity, nutrition, sun exposure and biological factors
Source: PLoS One. 2020 Sep 22;15(9):e0238127. doi: 10.1371/journal.pone.0238127 (PMC7508391; doi:10.1371/journal.pone.0238127)
Supplement: S1 Table — (DOCX) [file pone.0238127.s002.docx]

Table 2. Results of logistic regression analysis - odds ratios (OR) calculated for a Normal **BMD in the distal part** of the forearm bone

| Variable | | Low BMD n=91 | Normal  BMD n=409 | Univariate regression  models | Multivariate regression model^a^ |
| --- | --- | --- | --- | --- | --- |
|  |  |  |  | OR crude (95%CI) | OR adjusted  (95%CI) |
| **Place to live** | Small town | 46.2 | 50.9 | 1 | Not included  in the model |
|  | Agglomeration | 53.8 | 49.1 | 0.83 (0.53-1.31) |  |
| **BMI** | Norm | 36.7 | 37.3 | 1 | Not included  in the model |
|  | Overweight | 44.4 | 35.8 | 0.79 (0.47-1.32) |  |
|  | Obesity | 18.9 | 27.0 | 1.40 (0.74-2.65) |  |
| **Biological age** | Perimenopause | 3.3 | 31.1 | 1 | 1 |
|  | Premenopause | 4.4 | 20.3 | 0.49 (0.11-2.24) | 0.42 (0.09-2.03) |
|  | Postmenopause | 92.3 | 48.7 | 0.06** (0.02-0.18) | 0.10** (0.03-0.32) |
| **Calcium (mg/day)** | Deficiency | 96.7 | 83.1 | 1 | Not included  in the model |
|  | Recommended intake | 3.3 | 16.9 | 5.95* (1.83-19.4) |  |
| **Vitamin D (μg/day)** | Deficiency | 96.7 | 96.3 | 1 | Not included  in the model |
|  | Recommended intake | 3.3 | 3.7 | 1.12 (0.32-3.94) |  |
| **Osteoporosis in the family** | No | 82.4 | 85.0 | 1 | Not included  in the model |
|  | Yes | 17.6 | 15.0 | 0.82 (0.45-1.51) |  |
| **Past PA** | Inactive | 18.7 | 8.3 | 1 | Not included  in the model |
|  | Moderately active | 76.9 | 64.3 | 1.88 (0.99-3.56) |  |
|  | High | 4.4 | 27.4 | 14.0** (4.41-44.4) |  |
| **Present habitual PA** | Insufficient | 83.5 | 46.2 | 1 | 1 |
|  | Sufficient | 15.4 | 42.3 | 4.97** (2.71-9.11) | 2.38* (1.22-4.64) |
|  | High | 1.1 | 11.5 | 18.9* (2.56-139) | 7.95 (1.03-61.5) |
| **Past SA** | Insufficient | 35.2 | 9.5 | 1 | 1 |
|  | Sufficient | 64.8 | 90.5 | 5.15** (2.99-8.85) | 2.51* (1.37-4.58) |
| **Present SA** | Insufficient | 91.2 | 50.9 | 1 | 1 |
|  | Sufficient | 8.8 | 49.1 | 10.0** (4.73-21.2) | 4.39** (1.97-9.79) |

Legend: BMD – Bone mineral density; PA- physical activity; SA- sun exposure; a - R2 Nagelkerke for the multivariate regression model = 0.359; The statistical significance: *p<0.01 **<0.001
