# Supplementary material for: Determinants of bone health in adults Polish women: The influence of physical activity, nutrition, sun exposure and biological factors
Source: PLoS One. 2020 Sep 22;15(9):e0238127. doi: 10.1371/journal.pone.0238127 (PMC7508391; doi:10.1371/journal.pone.0238127)
Supplement: S2 Table — (DOCX) [file pone.0238127.s003.docx]

Table 3. Results of logistic regression analysis - odds ratios (OR) calculated for a Normal **BMD in the proximal part** of the forearm bone

| Variable | | Low BMD n=91 | Normal  BMD n=409 | Univariate regression  models | Multivariate regression model^a^ |
| --- | --- | --- | --- | --- | --- |
|  |  |  |  | OR crude (95%CI) | OR adjusted  (95%CI) |
| **Place to live** | Small town | 45.7 | 55.5 | 1 | Not included  in the model |
|  | Agglomeration | 54.3 | 44.5 | 0.68 (0.47-0.96) |  |
| **BMI** | Norm | 36.1 | 38.5 | 1 | 1 |
|  | Overweight | 41.1 | 32.6 | 0.74 (0.49-1.12) | 0.84 (0.45-1.55) |
|  | Obesity | 22.9 | 28.9 | 1.18 (0.75-1.86) | 2.68* (1.39-5.13) |
| **Biological age** | Perimenopause | 16.0 | 39.0 | 1 | 1 |
|  | Premenopause | 9.6 | 27.5 | 1.18 (0.66-2.10) | 0.97 (0.44-2.15) |
|  | Postmenopause | 74.5 | 33.5 | 0.18** 0.12-0.29 | 0.35** (0.19-0.63) |
| **Calcium (mg/day)** | Deficiency | 96.1 | 72.0 | 1 | 1 |
|  | Recommended intake | 3.9 | 28.0 | 9.57** (4.89-18.7) | 3.87** (1.69-8.89) |
| **Vitamin D (μg/day)** | Deficiency | 97.9 | 94.5 | 1 | Not included  in the model |
|  | Recommended intake | 2.1 | 5.5 | 2.68 (0.99-7.26) |  |
| **Osteoporosis in the family** | No | 79.4 | 91.3 | 1 | 1 |
|  | Yes | 20.6 | 8.7 | 0.37** (0.21-0.64) | 0.47 (0.22-0.97) |
| **Past PA** | Inactive | 16.7 | 1.8 | 1 | 1 |
|  | Moderately active | 77.3 | 52.8 | 6.20** (2.18-17.6) | 4.70 (1.28-17.3) |
|  | High | 6.0 | 45.4 | 68.4** (21.8-214) | 28.1** (6.78-116.7) |
| **Present habitual PA** | Insufficient | 72.3 | 28.0 | 1 | 1 |
|  | Sufficient | 23.4 | 55.5 | 6.13** (4.05-9.28) | 3.36** (1.95-5.77) |
|  | High | 4.3 | 16.5 | 10.0** (4.92-20.5) | 4.77** (2.03-11.2) |
| **Past SA** | Insufficient | 23.0 | 2.8 | 1 | 1 |
|  | Sufficient | 77.0 | 97.2 | 10.6 ** (4.49-24.9) | 3.22 (1.10-9.43) |
| **Present SA** | Insufficient | 79.8 | 30.3 | 1 | 1 |
|  | Sufficient | 20.2 | 69.7 | 9.09** (6.03-13.7) | 2.85** (1.68-4.85) |

Legend: BMD – Bone mineral density; PA- physical activity; SA- sun exposure; a - R2 Nagelkerke for the multivariate regression model = 0.359; The statistical significance: *p<0.01 **<0.001
